# Predicting Chronic Fine and Coarse Particulate Exposures Using Spatiotemporal Models for the Northeastern and Midwestern United States

**DOI:** 10.1289/ehp.11692

**Published:** 2008-11-19

**Authors:** Jeff D. Yanosky, Christopher J. Paciorek, Helen H. Suh

**Affiliations:** 1 Exposure, Epidemiology and Risk Program, Department of Environmental Health and; 2 Department of Biostatistics, Harvard School of Public Health, Boston, Massachusetts, USA

**Keywords:** air pollution, extinction coefficient, fine particulate matter, geographic information system, generalized additive mixed models, geostatistics, spatial smoothing, spatiotemporal modeling, visual range

## Abstract

**Background:**

Chronic epidemiologic studies of particulate matter (PM) are limited by the lack of monitoring data, relying instead on citywide ambient concentrations to estimate exposures. This method ignores within-city spatial gradients and restricts studies to areas with nearby monitoring data. This lack of data is particularly restrictive for fine particles (PM with aerodynamic diameter < 2.5 μm; PM_2.5_) and coarse particles (PM with aerodynamic diameter 2.5–10 μm; PM_10–2.5_), for which monitoring is limited before 1999. To address these limitations, we developed spatiotemporal models to predict monthly outdoor PM_2.5_ and PM_10–2.5_ concentrations for the northeastern and midwestern United States.

**Methods:**

For PM_2.5_, we developed models for two periods: 1988–1998 and 1999–2002. Both models included smooth spatial and regression terms of geographic information system-based and meteorologic predictors. To compensate for sparse monitoring data, the pre-1999 model also included predicted PM_10_ (PM with aerodynamic diameter < 10 μm) and extinction coefficients (km^−1^). PM_10–2.5_ levels were estimated as the difference in monthly predicted PM_10_ and PM_2.5_, with predicted PM_10_ from our previously developed PM_10_ model.

**Results:**

Predictive performance for PM_2.5_ was strong (cross-validation *R*^2^ = 0.77 and 0.69 for post-1999 and pre-1999 PM_2.5_ models, respectively) with high precision (2.2 and 2.7 μg/m^3^, respectively). Models performed well irrespective of population density and season. Predictive performance for PM_10–2.5_ was weaker (cross-validation *R*^2^ = 0.39) with lower precision (5.5 μg/m^3^). PM_10–2.5_ levels exhibited greater local spatial variability than PM_10_ or PM_2.5_, suggesting that PM_2.5_ measurements at ambient monitoring sites are more representative for surrounding populations than for PM_10_ and especially PM_10–2.5_.

**Conclusions:**

We provide semiempirical models to predict spatially and temporally resolved long-term average outdoor concentrations of PM_2.5_ and PM_10–2.5_ for estimating exposures of populations living in the northeastern and midwestern United States.

Chronic exposures to airborne particulate matter (PM) are related to increased mortality as well as lung cancer, ischemic heart disease, dysrhythmias, heart failure, and cardiac arrest (Abbey et al. 1999; Dockery et al. 1993; Finkelstein et al. 2003; Jerrett et al. 2005; Miller et al. 2007; Pope et al. 1995, 2002, 2004). These health effects have been shown for populations living near stationary ambient monitoring (SAM) sites and primarily and most consistently for exposure to PM with an aerodynamic diameter < 2.5 μm (PM_2.5_). It is possible that PM_2.5_, which originates from primary emissions from combustion sources and from secondary formation in the atmosphere, has different chronic health impacts than larger coarse particles (PM with aerodynamic diameters between 2.5 and 10 μm; PM_10–2.5_) because of their different composition. In contrast to PM_2.5_, PM_10–2.5_ is typically generated from mechanical grinding or crushing, as well as from windblown dust. The chronic health effects of PM_10–2.5_, however, have been little studied (Brunekreef and Forsberg 2005).

Exposure assessment in chronic PM studies has been severely limited by the lack of ambient monitoring data for PM_2.5_ and especially PM_10–2.5_, which are sparse over both time and space, particularly for periods before 1999. Because of this lack of data, some epidemiologic studies of PM_2.5_ have restricted their health effect analyses to populations near SAM sites (Dockery et al. 1993; Miller et al. 2007), whereas others have estimated exposures using concentrations averaged across metropolitan areas (Pope et al. 1995, 2002, 2004). Although this averaging may reduce instrument errors (and thereby classical exposure error), it ignores within-city spatial gradients in exposure, which can be substantial and may have important implications for health. For example, in a study conducted in Los Angeles, California, Jerrett et al. (2005) estimated chronic exposures, using PM_2.5_ data from one year (2000) and a spatial model that captured within-city spatial gradients, and showed that within-city gradients in PM_2.5_ were more strongly related to health effects than between-city gradients. These results were consistent with those of Miller et al. (2007), who also found larger effects for within-city comparisons. It is possible that health effects associated with chronic PM_2.5_ exposure are even larger: Miller et al. (2007) and Jerrett et al. (2005) assumed that spatial gradients in PM_2.5_ were constant over time, a simplifying assumption that may lead to bias (Haneuse et al. 2007).

To address these limitations, we estimated monthly PM_2.5_ and PM_10–2.5_ concentrations using spatiotemporal models that incorporated both small- and large-scale spatial trends, and that allowed these trends to change over time. These models were used to estimate PM_2.5_ and PM_10–2.5_ concentrations from 1988 through 2002 for populations living within the northeastern and midwestern United States.

## Methods

We predicted highly time- and space-resolved PM_2.5_ concentrations across our study region using geographic information system (GIS)-based spatiotemporal models of monthly PM_2.5_ concentrations. We did so for PM_2.5_ by developing and validating two models: one for 1988–1998 (pre-1999) and another for 1999–2002 (post-1999). Both models build on the GIS-based spatiotemporal model we developed for PM with an aerodynamic diameter < 10 μm (PM_10_) (Yanosky et al. 2008) and use publicly available air pollution, geographic, and meteorologic data. The pre-1999 model also used extinction coefficients derived from airport visibility data to predict location-specific outdoor PM_2.5_ concentrations. For PM_10–2.5_, we estimated spatially resolved monthly values by difference using the PM_2.5_ models and our previously developed PM_10_ model.

### *Data.* PM_2.5_ and PM_10–2.5_ data

**Figure 1:**
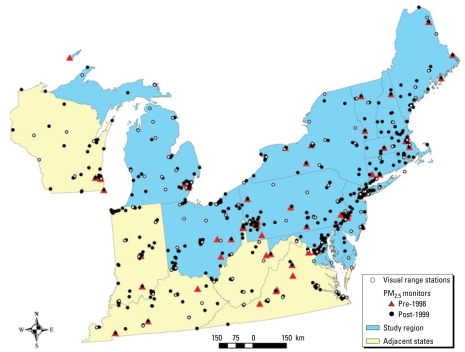
Map of the PM_2.5_ monitoring locations in the study region and adjacent states for monitoring sites reporting data from 1988 to 1998 and separately from 1999 to 2002. The locations of weather stations reporting visual range are also shown.

Outdoor PM_2.5_ concentration data from the northeastern and midwestern United States (the study region) were included in this analysis (Figure 1). Data collected in states adjacent to the study region were also included in the models to minimize boundary effects (Figure 1). PM_2.5_ data were obtained from the U.S. Environmental Protection Agency (EPA) Air Quality System (AQS) (U.S. EPA 2008); the Visibility Information Exchange Web System (2004) for Interagency Monitoring of Protected Visual Environments (IMPROVE), Stacked Filter Unit (a predecessor to IMPROVE), and Clean Air Status and Trends (CASTNet) networks; and Harvard research studies including the Twenty-four Cities Study and Five Cities Study (Spengler et al. 1996; Suh et al. 1997). The U.S. EPA AQS network provided about 88% of the PM_2.5_ concentration values and monitoring locations in the study region. Of the 546 monitoring locations, 386 were in the study region, with the remaining in adjacent states (Figure 1).

Monthly average PM_2.5_ concentrations were approximately log-normally distributed, with geometric mean and geometric standard deviation of 13.0 μg/m^3^ and 1.5, respectively, across the study region. Measured PM_10–2.5_ levels (estimated as the difference in measured monthly average PM_10_ and PM_2.5_ levels at co-located sites) were also approximately log-normally distributed, with geometric mean and geometric standard deviation of 6.9 μg/m^3^ and 2.44, respectively.

#### Geographic, meteorologic, and visibility data

Characteristics of the PM monitoring sites were quantified using a GIS (ArcMap 9; ESRI, Redlands, CA), including the following:

- Distance to nearest roadways for road classes A1 (primary roads, typically interstates, with limited access), A2 (primary major, non-interstate roads), A3 (smaller, secondary roads, usually with more than two lanes), and A4 (two-lane, typically surface roads used for local traffic) using ESRI StreetMap data;
- Urban land use (the proportion of low-intensity residential, high-intensity residential, and industrial/commercial/transportation land uses within 1 km) using data from the U.S. Geological Survey (USGS) National Land Cover Dataset (2004; Homer et al. 2004);
- Block group, tract, and county population density from the U.S. Census TIGER files from ESRI Data & Maps (ESRI);
- Point-source emissions of PM_2.5_ within circular buffers (1- and 10-km radii) and county-level area-source PM_2.5_ emissions from the U.S. EPA National Emissions Inventory (U.S. EPA 2005);
- Elevation from the USGS National Elevation Dataset (USGS 2005).

Monthly average temperature, wind speed, sea level-adjusted barometric pressure, and total precipitation were obtained from the National Climatic Data Center (2005) and spatially smoothed to the monitoring locations for each month. Additional details regarding these geographic and meteorologic covariates can be found in Yanosky et al. (2008).

Extinction coefficients were estimated using daily airport visual range observations (Faulke and Husar 1998; Ozkaynak et al. 1985) after correction for relative humidity and truncation, spatially smoothed to monitoring and prediction locations, then averaged by month (Paciorek et al. 2008).

### Statistical models

To compensate for the spatially sparse PM_2.5_ measurements before 1998, we constructed separate models for the pre-1999 and post-1999 time periods. Both models predicted PM_2.5_ concentrations using generalized additive mixed models (GAMMs), with bivariate penalized spline terms for space and one-dimensional penalized spline terms for GIS-based and meteorologic predictors.

#### Post-1999 PM_2.5_ spatiotemporal model

The structure of the post-1999 PM_2.5_ model followed that for the PM_10_ model (Yanosky et al. 2008):


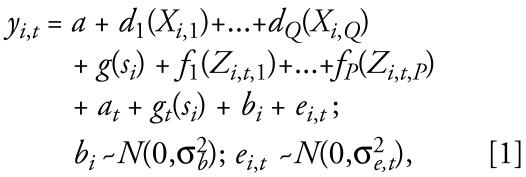


where *y_i,t_* is the natural log-transformed monthly average PM_2.5_ for *i* = 1…*I*, *I* = 499 sites and *t* = 1…*T*, *T* = 48 monthly time periods from 1999 to 2002, and *s_i_* the projected spatial coordinate pair for the *i*th location. The Albers Equal Area Conic USGS projection was used for all geographic data (ESRI). *g_t_*(*s_i_*) accounts for residual monthly spatial variability and *g*(*s_i_*) for time-invariant spatial variability. *Z_i,t_*_,1_ through *Z_i,t,P_* are time-varying covariates, X*_i,_*_1_ through X*_i,Q_* are time-invariant GIS-based covariates, and *a_t_* is a monthly intercept that controls for the mean across all sites. *d_1_* through *d_Q_* and *f_1_* through *f_P_* are one-dimensional penalized spline smooth functions for *Q* time-invariant and *P* time-varying covariates. Note that *b_i_* is a site-specific random effect, hence our characterization of the model as a GAMM.

The post-1999 model was fit in two stages: the first to estimate site-specific terms adjusting for time-varying covariates and residual spatial variability, and the second to model the site-specific terms using site-specific, time-invariant GIS-based predictors and residual time-invariant spatial variability. The form of the two-stage model was


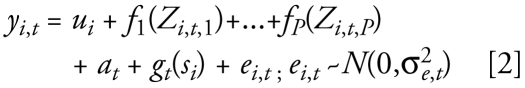



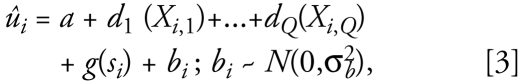


where *û_i_* is an estimated site-specific intercept that represents the adjusted long-term mean at each location.

The first stage (Equation 2) was fit iteratively in a back-fitting arrangement (Hastie and Tibshirani 1990) with *u_i_* + *f_1_()*…*f_p_()* estimated jointly and *a_t_* + *g_t_* (*s_i_*) estimated separately by month, such that variability in the concentrations is parsed between the covariates and the residual spatial terms in the first stage. The second stage (Equation 3) was fit to the estimated site-specific *û_i_* terms. Both stages were fit using calls to the gam() function (iteratively in the first stage) in the mgcv library of R (R Development Core Team 2008). Model predictions were obtained based on generating the covariates at locations of interest for each month and, once estimated, were transformed to the original scale by exponentiation.

#### Pre-1999 PM_2.5_ spatiotemporal model

The pre-1999 model assumed a relatively simple spatiotemporal structure because of the sparseness of PM_2.5_ data during the earlier time period. Specifically, seasonal spatial trends were assumed to be constant across years using the natural log-transformed ratio of PM_2.5_ to predicted PM_10_, which was preferable to alternative transformations (log-transformed PM_2.5_, ratio of PM_2.5_ to PM_10_, and logit-transformed PM_2.5_ to PM_10_ ratio) based on results from our exploratory models. Thus, the model described variation in the log-transformed ratio of PM_2.5_ to PM_10_, accounting for spatial and temporal variability in this ratio:


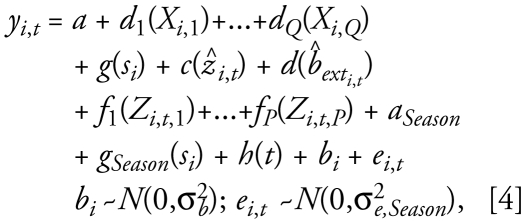


where *y_i_*_,_*_t_* = 1n(PM_2.5_/P̂M_10_) for *i* = 1…*I*, *I* = 546 sites and *t* = 1…*T*, *T* = 180 monthly time periods from 1988 to 2002, *ẑ_i, t_* is the predicted log PM_10_ concentration and *b̂_ext_i,t__* is the predicted log extinction coefficient after correction for relative humidity and truncation. *Season* has four levels (winter, spring, summer, and fall); *a_Season_* is a season-specific intercept, and *g_Season_*(*s_i_*) accounts for residual seasonal spatial variability. *h*(*t*) is a smoothly varying intercept that controls for the monthly mean across all sites. Also, *c* and *d* are one-dimensional penalized spline smooth functions. Otherwise, the notation is the same as for the post-1999 model.

The pre-1999 model was also fit in two stages:


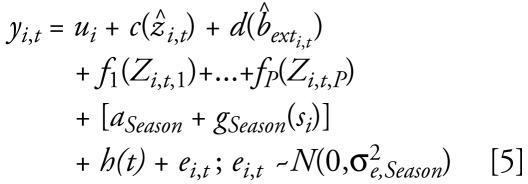



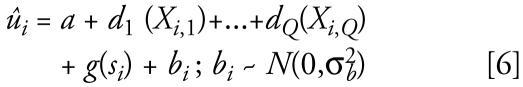


We obtained model predictions based on generating the covariates at locations of interest for each month, transforming the predicted PM_2.5_ to PM_10_ ratio back to the original scale by exponentiation, and then multiplying by predicted PM_10_.

#### Covariate selection

Covariates that were expected *a priori* to have a physical influence on PM_2.5_ levels were considered for inclusion in the post-1999 and pre-1999 models. Covariates remained in the model if their observed relationship with PM_2.5_ was consistent with known pollutant behavior (i.e., if the smooth functions were generally in the expected direction, analogous to slopes in the expected direction for linear terms) and if their inclusion improved predictive performance. Nonlinearity in the covariate effects was accounted for using spline terms, with at most 6 degrees of freedom considered sufficient to describe the general shape of each function.

**Figure 2:**
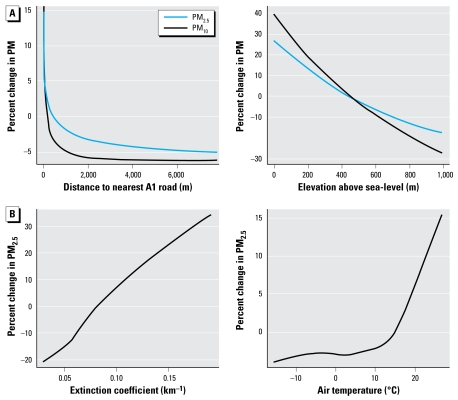
(*A*) Percent change in PM_2.5_ and PM_10_ as a function of distance to nearest interstate (A1 road) and elevation from the post-1999 PM_2.5_ model and the PM_10_ model presented by Yanosky et al. (2008), respectively. (*B*) Percent change in PM_2.5_ as a function of extinction coefficient and temperature from the pre-1999 PM_2.5_ model.

Using this procedure, we found several covariates to be important predictors in the post-1999 model, including distance to nearest A1 (Figure 2A) and A2 roads, urban land use, point-source PM_2.5_ emissions within 10 km, elevation (Figure 2A), wind speed, and precipitation. The covariate selection process contributed little to potential overfitting: The cross-validation *R*^2^ was comparable among locations used for the covariate selection process and those held out from this process (0.75 vs. 0.77, respectively). Important PM_2.5_ predictors for the pre-1999 model included predicted PM_10_ (from Yanosky et al. 2008), extinction coefficient (Figure 2B), wind speed, temperature (Figure 2B), precipitation, monthly time trend, distance to nearest A1 road, urban land use, block group and county population density, and elevation.

#### Model validation

We used cross-validation techniques both to inform covariate selection and to compare alternative model specifications. To evaluate the performance of the post-1999 model, we randomly selected monitoring sites from the 1999–2002 data and assigned them exclusively to 1 of 10 sets. Data from sets 1–9 (each set contains approximately 10% of the data) were held out in turn, with predictions generated at the locations of the held-out observations. Because the covariate selection process involved fitting multiple candidate models to the same data, we used set 10 to assess whether the covariate selection process contributed to overfitting, as reported above.

Similarly, for the pre-1999 model, monitoring sites from the 1988–1998 data were assigned exclusively to one of five sets at random. Data from sets one through five were held out in turn, with predictions generated at the locations of the held-out observations. Because 1988–1998 data were not spatially representative across the region, we performed a separate cross-validation step, where the pre-1999 model was used to predict at the held-out locations during 1999. Data from 1999 for a select group of monitors were included in the model-fitting data set to ensure that the number of monitoring locations and their population density in 1999 was similar to that from 1988–1998. Model overfitting due to covariate selection was not examined for the pre-1999 model because of the limited amount of PM_2.5_ data available between 1988 and 1998.

We determined the predictive abilities of the PM models using the squared Pearson correlation between the held-out observations and the model predictions (cross-validation *R*^2^), with both on the original rather than the log scale. We calculated prediction errors by subtracting held-out observations from the model predictions. Bias in model predictions was determined using the mean prediction error and the slopes from linear regression of the held-out values against the observations. We estimated the absolute precision of model predictions by taking the square root of the mean of the squared prediction errors (RMSPE), and relative precision by dividing the RMSPE by the arithmetic mean of the PM measurements in the appropriate size category. Bias and absolute precision of predicted PM_2.5_ levels from cross-validation were also evaluated by geographic location (state), urban land use and population density, season, monitoring network, and monitoring objective.

To evaluate the ability of our models to predict PM_10–2.5_, we compared monthly predicted PM_10–2.5_ values with PM_10–2.5_ measured values at sites where PM_2.5_ and PM_10_ monitors were co-located. We performed separate cross-validation procedures for PM_10–2.5_ by again holding out co-located PM_2.5_ and PM_10_ sites in 10 sets.

#### Sensitivity analyses

We compared the performance of our model with monthly varying spatial surfaces with alternative models with either four seasonal spatial terms or 16 season by year spatial terms. A smoothly varying intercept, *h*(*t*), where *t* = 1,…, *T*, *T* = 180, was added to both alternative models to allow for monthly control for the mean across all sites.

Additionally, we compared our post-1999 model with two simple spatial interpolation approaches, inverse distance weighting (IDW) and nearest neighbor (NN) interpolation. For the IDW approach, we used the squared inverse distance as the weighting function. For the NN approach, we excluded monitors that were not within 50 km of another monitor.

To evaluate the impact of the extinction coefficient term on the predictive ability of the pre-1999 model, we fit the model without this term. Additionally, we compared our pre-1999 model with a simplistic ordinary least squares linear regression model that assumes a single, fixed PM_2.5_ to PM_10_ ratio over space and time:


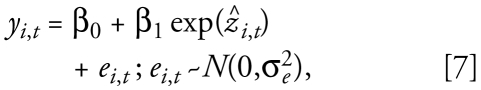


where *y_i,t_* is the monthly site average PM_2.5_ and exp(*ẑ_i,t_*) is the predicted PM_10_ concentration as in Equation 4.

### Data analysis

To evaluate the spatial heterogeneity in PM levels, we considered how PM levels at unmeasured locations (as best described by model predictions) differed on average from observed values by calculating mean squared deviations (MSDs) between PM levels measured at monitoring sites and PM levels predicted at unmeasured locations on an 8-km grid over the study region, doing this separately for PM_2.5_, PM_10_, and PM_2.5–10_. Only monitoring sites that were within a metropolitan statistical area (MSA) and those in the AQS network with population exposure as their monitoring objective were included in this analysis. PM_10_ predictions were made using our previously developed model for PM_10_ (Yanosky et al. 2008). As an estimate of the amount of spatial variability at each distance relative to the total spatial variability at the regional scale, we divided MSDs at distances < 400 km by the median MSD at 400 km for each pollutant. We then used the medians and 25th and 75th percentiles within 10-km bins to describe trends in spatial variability as a function of distance (i.e., the variogram). Only monthly predictions from 1999 to 2002 for PM_2.5_, PM_10_, and PM_10–2.5_ were included in this analysis, because pre-1999 PM_2.5_ predictions were based on predicted PM_10_ levels.

## Results

We first present results describing the predictive performance of the PM_2.5_ and PM_10–2.5_ models. We then present results from our sensitivity analyses, comparing these PM_2.5_ models to alternative models. Finally, we present results from our evaluation of the spatial heterogeneity of PM_2.5_, PM_10_, and PM_10–2.5_ levels across metropolitan areas.

### Model predictive performance

Model fit, cross-validation, and regression results for the post-1999 and pre-1999 PM_2.5_ models are presented in Table 1. Both PM_2.5_ models performed well. The post-1999 PM_2.5_ model explained most of the variability in monthly measured PM_2.5_ levels (model fit *R*^2^ = 0.87 and cross-validation *R*^2^ = 0.77). The pre-1999 PM_2.5_ model also performed well (cross-validation *R*^2^ = 0.68 and 0.69 using 1988–1998 and 1999 cross-validation data, respectively). For both models, results from linear regression showed good agreement between measured values and model predictions, with slopes near 1 and intercepts near 0.

Both the post-1999 and pre-1999 PM_2.5_ models predicted PM_2.5_ concentrations with little bias (−0.2 and −0.3 μg/m^3^, respectively) and high absolute and relative precision (2.2 and 2.7 μg/m^3^ and 16.7% and 15.4%, respectively). Both PM_2.5_ models performed well across the region and also in both rural and urban areas, although each had slightly better precision in spring and fall seasons compared with summer and winter [see Supplemental Material, Table 1 (online at http://www.ehponline.org/members/2008/11692/suppl.pdf)].

Our PM_2.5_ models and our previous PM_10_ model (Yanosky et al. 2008) relied on similar predictors, all including elevation, urban land use, distance to nearest A1 road, wind speed, and precipitation. Further, smooth functions of these predictors common to each model were generally similar in shape (Figure 2A). For example, PM_2.5_ and PM_10_ levels decreased similarly from 0 to about 250 m away from the nearest A1 road, indicating that this term captured micro-scale (0–100 m) and middle-scale (100–250 m) gradients in PM_2.5_ and PM_10_ from traffic sources similarly. Beyond about 400 m, however, PM_2.5_ and PM_10_ showed a slightly different relation with distance to A1 roads, as predicted PM_10_ levels decreased more quickly than PM_2.5_ levels. This modest difference is likely attributable to the greater relative contribution of secondary aerosols to PM_2.5_, resulting in a more homogenous spatial distribution of PM_2.5_ than PM_10_. These findings suggest that distance to A1 road described neighborhood-scale (500 m–4 km) and urban-scale (4–7 km) gradients slightly better for PM_2.5_ than for PM_10_.

**Figure 3:**
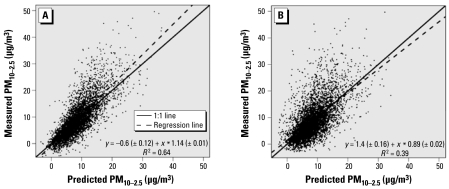
Scatter plot of monthly predicted versus measured PM_10–2.5_ concentrations in the northeastern and midwestern United States from 1999 to 2002 (*A*) including all measured locations and (*B*) from cross-validation.

Our models performed less well in estimating PM_10–2.5_ than PM_2.5_. With post-1999 data, measured PM_10–2.5_ values were generally centered around the predicted PM_10–2.5_ values (Figure 3A), with little proportional bias (slope of 1.14) and much of the variability in the measurements explained by the predictions (*R*^2^ = 0.64). From cross-validation (Figure 3B), predicted PM_10–2.5_ levels again exhibited little bias (slope = 0.89), but predictive performance was poorer (cross-validation *R*^2^ of 0.39). The absolute and relative precision of predicted PM_10–2.5_ levels from cross-validation was relatively low (5.5 μg/m^3^ and ± 61.5%, respectively). Predictive performance for pre-1999 data was comparable with that for post-1999 data (cross-validation *R*^2^ = 0.33).

Predictive performance of the PM models improved substantially when long-term, multiyear averages (rather than monthly averages) of measured PM levels and model predictions (one mean value per site for measurements and predictions) were compared (cross-validation *R*^2^ = 0.81 and 0.75 for post-1999 and pre-1999 PM_2.5_ models, respectively, and 0.63 and 0.65 for post-1999 and pre-1999 PM_10–2.5_ excluding one site in northern Maine, respectively). These long-term average results are for sites with at least 39 of 48 months of valid data for the post-1999 models, and at least 10 of 12 months of valid data (using 1999 cross-validation data) for the pre-1999 models.

### Sensitivity analyses

Model fit, cross-validation, and regression results for alternative models are presented in Table 1. Our post-1999 and pre-1999 PM_2.5_ models were preferable to alternative models with different spatiotemporal structures. The post-1999 model with monthly spatial terms performed better than simpler models with four seasonal or 16 season by year spatial terms (Table 1; cross-validation *R*^2^ = 0.77 for the post-1999 model vs. 0.68 and 0.72 for the alternative models, respectively), which demonstrates the improvement in predictive performance gained by modeling space-time interaction at the monthly rather than the seasonal level. Further, the post-1999 model performed better than simple spatial interpolation methods (cross-validation *R*^2^ = 0.60 for IDW and 0.61 for NN). With a matched data set, which addressed the fact that fewer data points were available for NN analysis (5,210 instead of 10,444), the performance of our post-1999 model performance remained high (cross-validation *R*^2^ = 0.76) and was comparable with that across all observations. Additionally, the simple spatial interpolation methods exhibited greater proportional bias than the post-1999 model, as indicated by slopes from linear regression of 0.92 and 0.77 for IDW and NN, respectively, versus 0.95 for the post-1999 model (Table 1).

Although the performance of the pre-1999 model with the extinction coefficient term was highly comparable with that without (Table 1; cross-validation *R*^2^ = 0.69 with extinction coefficient and 0.70 without, both using 1999 cross-validation data), the extinction coefficient was included in the final model, as its smooth term indicated a nearly linear increase in PM_2.5_ levels with higher extinction coefficients (Figure 2B), a result consistent with light extinction theory (Ozkaynak et al. 1985). However, likely because of the truncation in airport visibility data, the distribution of the extinction coefficients was quite narrow, with 25th and 75th percentiles of 0.06 and 0.07 km^−1^, respectively, leading to little predictive power in this covariate. We also noted considerable changes in the seasonal smooth spatial surfaces in pre-1999 PM_2.5_ models with and without the extinction coefficient term. This may explain the lack of improvement in predictive performance when extinction coefficients were included in the model.

The alternative Pre-1999 model that assumed a fixed PM_2.5_ to PM_10_ ratio over time and space also exhibited lower predictive performance than the pre-1999 model (Table 1; cross-validation *R*^2^ = 0.53 vs. 0.69, respectively), which demonstrates the improvement in predictive performance from allowing spatial trends in the PM_2.5_ to PM_10_ ratio to change seasonally and from accounting for temporal trends in this ratio.

### PM concentrations across the study Region

**Figure 4:**
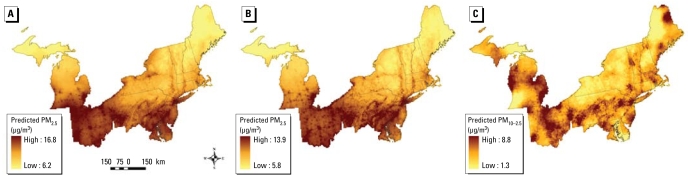
Maps of mean predicted concentrations for (*A*) PM_2.5_ across all months from 1988 to 1998 from the pre-1999 model, (*B*) PM_2.5_ across all months from 1999 to 2002 from the post-1999 model, and (*C*) PM_10–2.5_ across all months from 1999 to 2002, estimated as the difference in monthly predictions of PM_10_ and PM_2.5_. Low and high values are 5th to 95th percentiles, respectively.

Maps of the predicted PM_2.5_ concentrations averaged across all months from 1988 to 1998 are shown in Figure 4A and from 1999 to 2002 in Figure 4B. Both maps show consistent spatial patterns, with predicted PM_2.5_ concentrations highest in eastern Ohio, Pennsylvania, and western Maryland and in urban areas such as New York City, New York, and Detroit, Michigan. Predicted PM_2.5_ levels are lowest in northern areas of Maine, New York, and Michigan. As shown in Figure 4C, mean predicted PM_10–2.5_ concentrations (1999–2002) have a substantially different spatial pattern, with considerably more spatial heterogeneity in PM_10–2.5_ levels compared with PM_2.5_.

**Figure 5:**
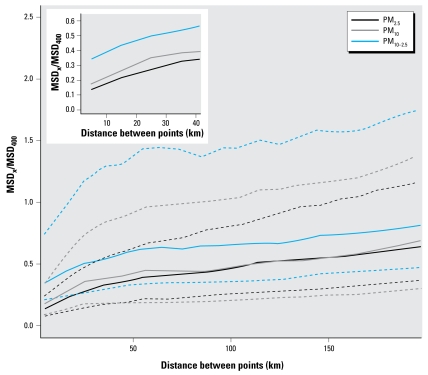
The proportion of local spatial variability relative to the total (MSD_x_/MSD_400_) as a function of distance for PM_2.5_, PM_10_, and PM_10–2.5_ from 1999–2002 for AQS population exposure monitors within MSAs. Solid lines are medians and dotted lines are 25th and 75th percentiles. The inset shows the medians from 0 to 40 km in greater detail.

Spatial trends in predicted PM_2.5_ concentrations were relatively similar to those found for PM_10_ (Yanosky et al. 2008). However, PM_2.5_ concentrations were more spatially uniform compared with PM_10_ and especially with PM_10–2.5_. Figure 5 shows trends in the proportion of local spatial variability relative to the total at 400 km as a function of the distance between PM monitoring locations and prediction grid points for PM_2.5_, PM_10_, and PM_10–2.5_. At distances < 75 km, this proportion was generally lower and less variable for PM_2.5_ than for PM_10_ or PM_10–2.5_ (Figure 5), consistent with the greater contribution of secondary particles (such as sulfate) for PM_2.5_ compared with PM_10_ or PM_10–2.5_ (Allen and Turner 2008).

Despite this, at even moderately close distances (between 0 and 25 km) relative to the scale of a typical metropolitan area, PM_2.5_, PM_10_, and especially PM_10–2.5_ exhibited substantial spatial heterogeneity as evidenced by the sharp increase in the 75th percentiles (Figure 5) of the MSD_x_/MSD400, suggesting the importance of within-city gradients in PM levels. Further, these spatial gradients were larger for PM_10_ and PM_10–2.5_ than for PM_2.5_, as the 75th percentiles for PM_10_ and PM_10–2.5_ increased more quickly than those for PM_2.5_, although medians for PM_10_ were only slightly higher than for PM_2.5_. Local, neighborhood-scale spatial variability (MSD_4 km_) constituted 7.8% of the total, regional scale variability (MSD_400 km_) for PM_2.5_, compared with 15.8% and 35.1% for PM_10_ and PM_10–2.5_, respectively, corresponding to the values at the leftmost part of the *x*-axis in Figure 5. Additionally, using a standard sums of squares decomposition, the proportions of spatial, temporal, and spatiotemporal variability in predicted PM_2.5_ levels were 50%, 32%, and 18%, respectively, compared with 41%, 23%, and 36% for PM_10–2.5_, respectively, indicating that monthly spatiotemporal changes are more important for PM_10–2.5_ than for PM_2.5_. Together, results indicate that PM_2.5_ levels were more spatially homogenous than PM_10_ and especially PM_10–2.5_ levels.

When these within-MSA population exposure monitoring sites were stratified by urbanization (above or below the median proportion of urban land use within 1 km and county-level population density of the monitoring site), PM_2.5_, PM_10_, and PM_10–2.5_ levels were more spatially heterogeneous in highly urbanized areas compared with less urbanized areas. Further, the amount of spatial heterogeneity in PM levels varied by season, likely because PM_2.5_ represents a smaller proportion of PM_10_ in the spring and fall compared with winter and summer. In spring and fall, PM_2.5_ concentrations were more spatially homogeneous than PM_10_ and especially PM_10–2.5_, as evidenced by seasonal variograms and by the number of degrees of freedom in the seasonal spatial terms of the pre-1999 PM_2.5_ model (38 and 5 for spring and fall, and 355 and 303 for winter and summer, respectively). However, in winter and to a lesser extent summer, PM_2.5_ and PM_10_ exhibited similar levels of spatial variability, with both again less variable than PM_10–2.5_. These results suggest that spatial variability in PM_10_ in summer and especially in winter is affected primarily by variability in PM_2.5_, likely because of winter wood smoke emissions and differing meteorologic conditions across seasons. Results are consistent with greater mixing of the atmosphere in nonwinter months, with increased formation of secondary particles in summer.

## Discussion

By including location-specific covariates, our spatiotemporal models provide highly spatially and temporally resolved estimates of outdoor PM concentrations that can be used to estimate chronic exposures to PM_2.5_ and, for the first time, to PM_10–2.5_ as well. Importantly, our models allow spatial trends in predicted monthly average PM_2.5_ and PM_10–2.5_ concentrations to change over time, and therefore are able to provide realistic, accurate, and precise monthly location-specific predictions of PM levels that may be used as measures of chronic exposure. These values can be averaged to provide exposure measures for different time periods relevant to chronic health impacts (e.g., previous month, previous 3 months, previous year). As demonstrated by their satisfactory predictive ability, the models are able to predict PM_2.5_ and PM_10–2.5_ levels over large regions while maintaining within-city spatial gradients using location-specific covariates and spatial smoothing. We demonstrate for the first time a methodology for estimating chronic exposures to ambient PM_10–2.5_ over long time periods and across the northeastern and midwestern United States.

Our study represents a significant improvement over previous methods of estimating long-term average ambient PM concentrations, including the nearest air pollutant monitor approach and citywide or countywide averaging. By using spatial smoothing to perform local spatial averaging, our models reduce the potential for measurement error in estimates of chronic exposure to ambient PM, as occurs with the nearest neighbor approach, while maintaining within-city spatial gradients. Our post-1999 PM_2.5_ model also provided improved performance and exhibited less proportional bias (i.e., regression slope closer to 1) than interpolation methods. However, given their relatively strong performance and their simplicity, interpolation methods may be a reasonable alternative to more complicated spatiotemporal modeling of PM_2.5_ when resources are limited.

Our approach to model PM_2.5_ concentrations separately by time period (post-1999 and pre-1999) allowed monthly PM_2.5_ concentrations to be predicted for 15 years, including years before 1999 when PM_2.5_ data were extremely sparse. PM_2.5_ models for both time periods performed well by season and population density, as demonstrated using cross-validation techniques, albeit with slightly lower precision in some states and in winter and summer seasons. Results from both models demonstrated the importance of accounting for spatial variability, temporal variability, and location-specific covariates.

Model and spatiotemporal structure differed substantially between the pre- and post-1999 models, however, because of differences in the amount of available PM_2.5_ monitoring data. The pre-1999 model used predicted PM_10_ levels from the model described by Yanosky et al. (2008) to estimate PM_2.5_ to PM_10_ ratios. Although temporal trends in these ratios were adjusted for, this model assumed a fixed spatial trend in the ratio across years for each season (e.g., spatial trends in the ratio in winter 1988 were assumed to be the same as those in winter 1989). This innovative modeling approach leveraged the available information on PM_10_, extinction coefficients, and available PM_2.5_ data while allowing for spatially and temporally varying calibrations between PM_2.5_ and PM_10_ levels. In comparison, given the richer data set, the post-1999 PM_2.5_ model could rely on measured PM_2.5_ without PM_10_ and was more flexible, allowing spatial trends in PM_2.5_ to change monthly.

Our models are similar in structure to land-use regression models in that they incorporate the effects of GIS-based predictors. However, our models are distinct from typical land-use regression models in that they account for residual spatial variability using spatial smoothing and allow these spatial surfaces to change over time. Further, because they do not involve spatial modeling, typical land-use regression analyses are only applicable for small regions (across a city or metropolitan area), whereas our model is applicable to much larger areas (i.e., the entire northeastern and midwestern United States). Results from our analyses showing the importance of GIS-based predictors were consistent with those of Brauer et al. (2003). There the authors used GIS-based predictors in multiple linear regression models (but not spatial smoothing techniques) to explain variability in annual PM_2.5_ concentrations in the three areas: the Netherlands; Munich, Germany; and Stockholm County, Sweden. Brauer et al. reported RMSPE values between 1.1 and 1.6 μg/m^3^ for annual averages. Even though our models were based on monthly averages, included spatiotemporal modeling in addition to GIS predictors, and were applied to a larger domain (the northeastern and midwestern United States as opposed to three small areas in Europe), our model performance results were similar. We found only slightly higher RMSPE values of 2.2 and 2.7 μg/m^3^ for the post-1999 and pre-1999 models, respectively, for our monthly average PM_2.5_ predictions.

Predictive performance was lower for PM_10–2.5_ than for PM_2.5_ because *a*) fewer monitoring sites were available (only those with co-located PM_10_ and PM_2.5_ provide measured values), *b*) prediction errors were compounded by estimating PM_10–2.5_ by difference, and *c*) the PM_10_ model contained little highly spatially resolved information on PM_10–2.5_ sources, such as fugitive dust emissions and unpaved roads. In addition, relative precision estimates were worse for PM_10–2.5_ than for PM_2.5_ because typical PM_10–2.5_ concentrations are lower than those for PM_2.5_. However, predictive performance improved substantially when long-term, multiyear averages were considered.

Results from our analysis of the spatial heterogeneity of PM levels indicated that PM_2.5_ had the smallest amount of local spatial variability, followed by PM_10_, and finally by PM_10–2.5_. Further, they suggest that PM_2.5_ measurements at a SAM site are more representative for surrounding populations than for PM_10_, and more representative for PM_10_ than for PM_10–2.5_. However, spatial variability for the three pollutants increased nearly linearly at distances from 0 to about 50 km (though slightly less steeply between 25 and 50 km), suggesting that there was no fixed distance within which PM concentrations were entirely uniform within a metropolitan area. Together, these results suggest that epidemiologic analyses that compare the effects of PM_2.5_ with PM_10–2.5_ using only measurements at SAM sites as indicators of exposure should be interpreted with caution because of greater exposure prediction error for PM_10–2.5_ than for PM_10_ or PM_2.5_ (Zeger et al. 2000).

Our modeling approach assumes isotropy and stationarity of the spatial surfaces, normality and homoscedasticity of residuals, independence of the time-varying spatial terms, uniform effects of GIS-based and meteorologic covariates across space, and no interactions of these covariates; see Paciorek et al. (2008) for evidence supporting these simplifying assumptions.

The satisfactory performance of our two models demonstrates their suitability for estimating long-term average population exposures to ambient PM concentrations. These models have the potential to reduce exposure measurement error in air pollution health effect studies. We are currently using these models to estimate chronic PM_2.5_ and PM_10–2.5_ exposures in the Nurses’ Health Study, a large prospective cohort study of U.S. women, to examine the chronic health impacts of these pollutants.

## Figures and Tables

**Table 1:** Model fit, cross-validation, and regression results for the post-1999 and pre-1999 PM_2.5_ models.

Time period	Model description	CV data	No. of spatial terms^a^	Covariates included	CV results			
					Model fit *R*^2^^b^	Intercept^c^	Slope^c^	CV R^2^
Post-1999 (1999–2002)	Final model	1999–2002	48 month by year^d^	Full set	0.85	0.8 ± 0.07	0.95 ± 0.01	0.77
	Alternative season by year spatial terms	1999–2002	16 season by year^d^	Full set	0.78	0.3 ± 0.08	1.00 ± 0.01	0.72
	Alternative seasonal spatial terms	1999–2002	4 seasonal^d^	Full set	0.76	0.5 ± 0.09	0.99 ± 0.01	0.68
	IDW	1999–2002	None	None	—	0.61 ± 0.11	0.92 ± 0.01	0.60
	NN	1999–2002^e^	None	None	—	3.0 ± 0.13	0.77 ± 0.01	0.61
Pre-1999 (1988–1998)	Final model	1988–1998^f^	4 seasonal^d^	Full set	0.76	−0.4 ± 0.33	1.05 ± 0.02	0.68
		1999^g^	4 seasonal^d^	Full set	0.76	1.09 ± 0.22	0.94 ± 0.02	0.69
	Final model without extinction coefficient	1999^g^	4 seasonal^d^	Full set minus extinction coefficient	0.76	1.19 ± 0.21	0.94 ± 0.02	0.70
	Alternative fixed ratio model	1999^g^	None	None	0.61	1.5 ± 0.29	0.85 ± 0.02	0.53

CV, cross-validation.

a Corresponds to the extent of control for space–time interaction in the model.

b Using data in the study region only.

c Presented as parameter estimate ± SE from linear regression of held-out observations on predictions.

d Number of time-varying spatial terms fit in the first stage of the model in addition to one spatial term fit in the second stage.

e Only 5,210 observations available for comparison versus 10,444 observations for other models; excluded monitors did not have another monitor within 50 km.

f One observation in Ohio and all observations from one site in New York excluded as outliers.

g Four observations at one site in New York excluded as outliers.

## References

[b1-ehp-117-522] Abbey D, Nishino N, McDonnell W, Burchette R, Knutsen S, Beeson W (1999). Long-term inhalable particles and other air pollutants related to mortality in nonsmokers. Am J Respir Crit Care Med.

[b2-ehp-117-522] Allen D, Turner J (2008). Transport of atmospheric fine particulate matter: Part 1. Findings from recent field programs on the extent of regional transport within North America. J Air Waste Manag Assoc.

[b3-ehp-117-522] Brauer M, Hoek G, van Vliet P, Meliefste K, Fischer P, Gehring U (2003). Estimating long-term average particulate air pollution concentrations: application of traffic indicators and geographic information systems. Epidemiology.

[b4-ehp-117-522] Brunekreef B, Forsberg B (2005). Epidemiological evidence of effects of coarse airborne particles on health. Eur Respir J.

[b5-ehp-117-522] Dockery D, Pope CI, Xu X, Spengler J, Ware J, Fay M (1993). An association between air pollution and mortality in six US cities. N Engl J Med.

[b6-ehp-117-522] Faulke S, Husar R (1998). Maps of PM_2.5_ over the U.S. derived from regional PM_2.5_ and surrogate visibility and PM_10_ monitoring data. http://capita.wustl.edu/CAPITA/DataSets/FM_VISIB/FMVISIB.HTML.

[b7-ehp-117-522] Finkelstein M, Jerrett M, DeLuca P, Finkelstein N, Verma D, Chapman K (2003). Relation between income, air pollution and mortality: a cohort study. Can Med Assoc J.

[b8-ehp-117-522] Haneuse S, Wakefield J, Sheppard L (2007). The interpretation of exposure effect estimates in chronic air pollution studies. Stat Med.

[b9-ehp-117-522] Hastie T, Tibshirani R (1990). Generalized Additive Models.

[b10-ehp-117-522] Homer C, Huang C, Yang L, Wylie B, Coan M (2004). Development of a 2001 national landcover database for the United States. Photogrammetric Engineering and Remote Sensing.

[b11-ehp-117-522] Jerrett M, Burnett R, Ma R, Pope CI, Krewski D, Newbold K (2005). Spatial analysis of air pollutions and mortality in Los Angeles. Epidemiology.

[b12-ehp-117-522] Miller K, Siscovick D, Sheppard L, Shepherd K, Sullivan J, Anderson G (2007). Long-term exposure to air pollution and incidence of cardiovascular events in women. N Engl J Med.

[b13-ehp-117-522] National Climatic Data Center (2005). Home page.

[b14-ehp-117-522] Ozkaynak H, Schatz A, Thurston G, Isaacs R, Husar R (1985). Relationships between aerosol extinction coefficients derived from airport visual range observations and alternative measures of airborne particle mass. J Air Pollut Control Assoc.

[b15-ehp-117-522] Paciorek C, Yanosky J, Suh H (2008). Practical large-scale spatio-temporal modeling of particulate matter concentrations. Ann Appl Stat.

[b16-ehp-117-522] Pope CA, Burnett R, Thun M, Calle E, Krewski D, Ito K (2002). Lung cancer, cardiopulmonary mortality, and long-term exposure to fine particulate air pollution. JAMA.

[b17-ehp-117-522] Pope CA, Burnett R, Thurston G, Thun M, Calle E, Krewski D (2004). Cardiovascular mortality and long term exposure to particulate air pollution. Circulation.

[b18-ehp-117-522] Pope CA, Thun M, Namboodiri M, Dockery D, Evans J, Speizer F (1995). Particulate air pollution as a predictor of mortality in a prospective study of U.S. adults. Am J Respir Crit Care Med.

[b19-ehp-117-522] R Development Core Team (2008). R: A language and environment for statistical computing.

[b20-ehp-117-522] Spengler J, Koutrakis P, Dockery D, Raizenne M, Speizer F (1996). Health effects of acid aerosols on North American children: air pollution exposures. Environ Health Perspect.

[b21-ehp-117-522] Suh H, Nishioka Y, Allen G, Koutrakis P, Burton R (1997). The Metropolitan Acid Aerosol Characterization Study: results from the summer 1994 Washington, D.C. field study. Environ Health Perspect.

[b22-ehp-117-522] U.S. EPA (2005). U.S. Environmental Protection Agency National Emissions Inventory.

[b23-ehp-117-522] U.S. EPA (2009). U.S. Environmental Protection Agency Air Quality System.

[b24-ehp-117-522] USGS (2004). U.S. Geological Survey National Land Cover Dataset.

[b25-ehp-117-522] USGS (2005). U.S. Geological Survey National Elevation Dataset.

[b26-ehp-117-522] Visibility Information Exchange Web System (2004). Home page.

[b27-ehp-117-522] Wood S (2006). Generalized Additive Models: An Introduction with R.

[b28-ehp-117-522] Yanosky J, Paciorek C, Schwartz J, Laden F, Puett R, Suh H (2008). Spatio-temporal modeling of chronic PM_10_ exposure for the Nurses’ Health Study. Atmos Environ.

[b29-ehp-117-522] Zeger S, Thomas D, Dominici F, Samet J, Schwartz J, Dockery D (2000). Exposure measurement error in time-series studies of air pollution: concepts and consequences. Environ Health Perspect.

